# Supplementary material for: TF/PAR2 Signaling Axis Supports the Protumor Effect of Neutrophil Extracellular Traps (NETs) on Human Breast Cancer Cells
Source: Cancers (Basel). 2023 Dec 19;16(1):5. doi: 10.3390/cancers16010005 (PMC10778307; doi:10.3390/cancers16010005)
Supplement: Supplementary file 1 [file cancers-16-00005-s001.zip › cancers-2616469-supplementary.pdf]

Supplementary Materials

Supplementary Table S1. qPCR primer sequences.

Primer name	Forward primer (5′–3′)	Reverse primer (5′–3′)	Size bp
GAPDH	5′- TGCACCACCAACTGCTTAGG -3′	5′- GGCATGGACTGTGGTCATGAG -3′	87
IL1B	5′- GGACAGGATATGGAGCAACAA -3′	5′- TCTTTCAACACGCAGGACAG -3′	128
IL6	5′- TACCCCAGGAGAAGATTCC - 3′	5′- TTTTCTGCCAGTGCCTCTTT -3′	174
CXCL8	5′- CTGGACCCCAAGGAAAACTG -3′	5′- GAATTCTCAGCCCTCTTCAAAAAC -3′	65
MMP9	5′- GCAATGCTGATGGGAAACCC -3′	5′- AGAAGCCGAAGAGCTTGTCC -3′	144
ZEB1	5′- TGGAATGTATGCTTGTGATTTGTG -3′	5′- GAATAAGACCCAGAGTGTGAGAAG -3	225
CD44	5′- GGAGCAGCACTTCAGGAGGTTAC -3′	5′- GGAATGTGTCTTGGTCTCTGGTAGC -3′	129
PTGS2	5′- TGGTGCCTGGTCTGATGATG -3′	5′- GCCTGCTTGTCTGGAACAAC -3′	120

Supplementary Figure S1.

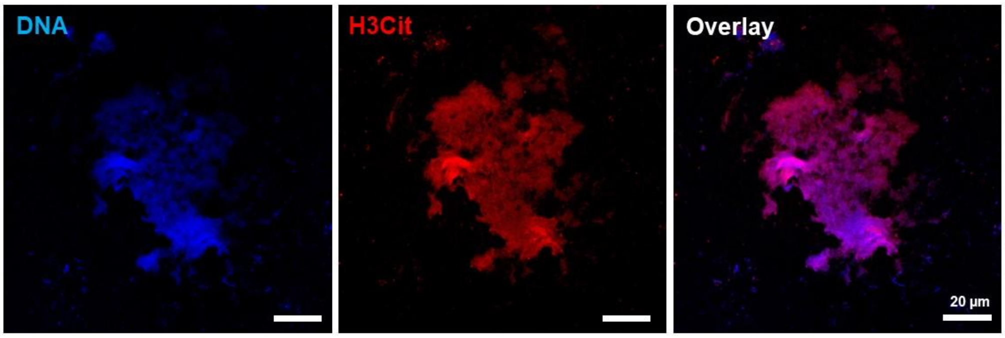

Supplementary Figure S1. Characterization of isolated NETs with DAPI and immunostaining with anti-citrullinated Histone 3. Isolated NETs were submitted to DNA staining with DAPI which correlates with citrullinated Histone 3 staining employing a rabbit polyclonal anti-histone H3 (citrulline R2 + R8 + R17) (ab5103, Abcam, MA, USA).

Supplementary Figure S2.

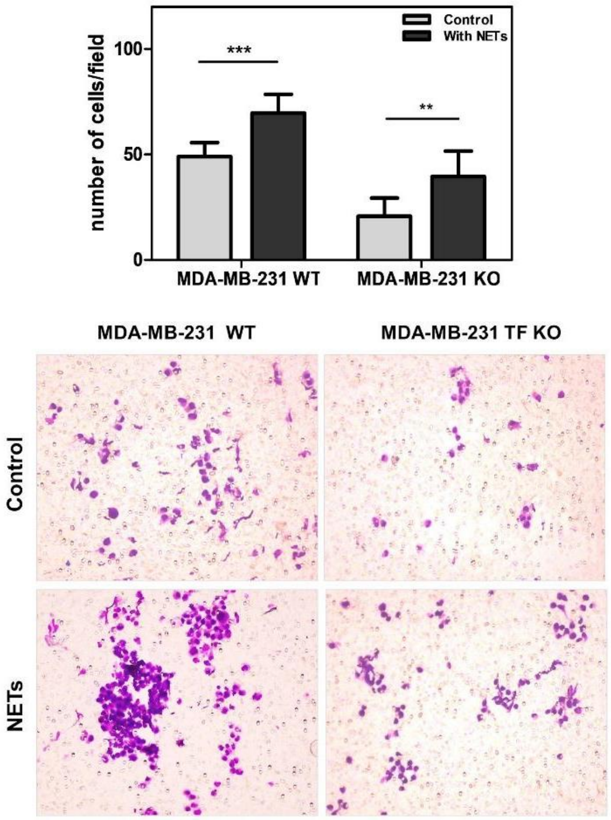

Supplementary Figure S2. NETs trigger TF-independent migratory capacity. Migration assays were performed as described by Martins-Cardoso et al. (37). MDA-MB-231 cells TF knockout (KO) or empty vector (WT) were treated with NETs (500 ng/mL) for 24 h. 5×10^4 cells were seeded in the upper well. DMEM medium supplemented with 2% FBS was used as a chemoattractant. After 6 hours of incubation, an 8 µm pore membrane was stained, and 10 fields per condition were photographed and counted. Values represent the mean ± standard deviation of three independent experiments, ** *p-value* < 0.01, *** *p-value* < 0.001, and *ns* = no significance (unpaired t-test).

Supplementary Figure S3.

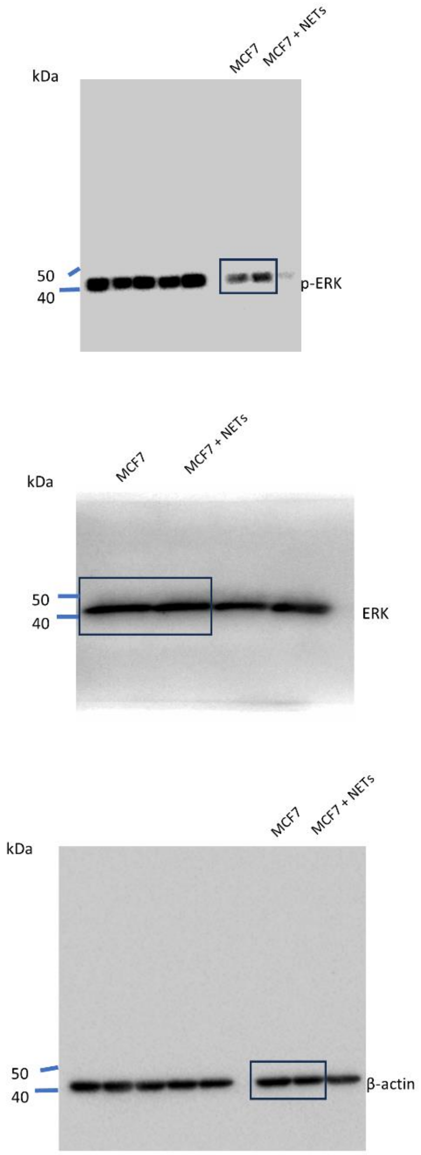

Supplementary Figure S3. Uncropped blots of Figure 3A; pERK/ERK staining represented in Figure 3.

Supplementary Figure S4.

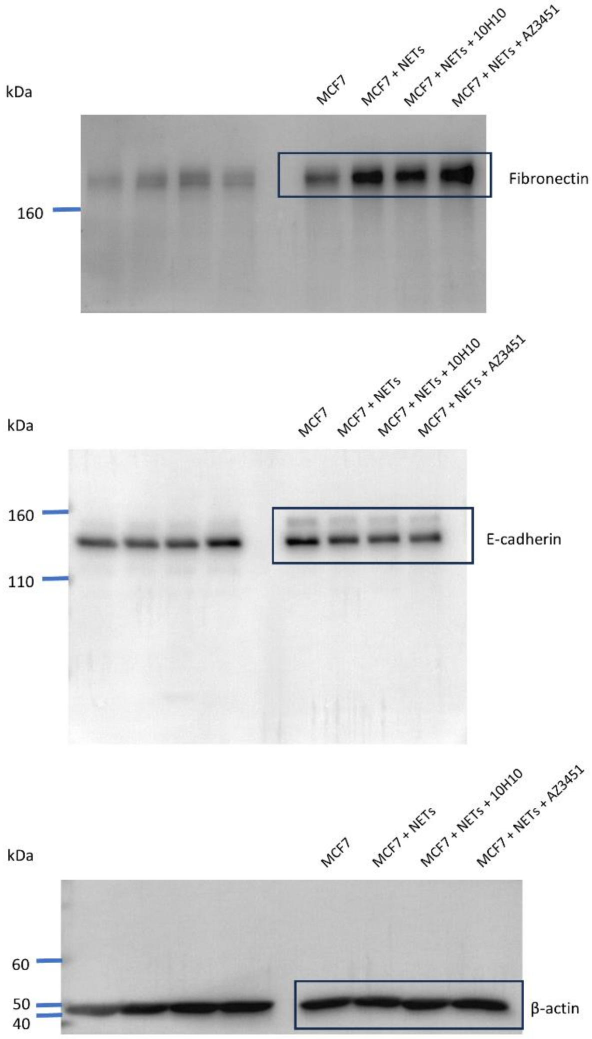

Supplementary Figure S4. Uncropped blots of Figure 7; Fibronectin and E-cadherin staining for MCF7 cells.

Supplementary Figure S5.

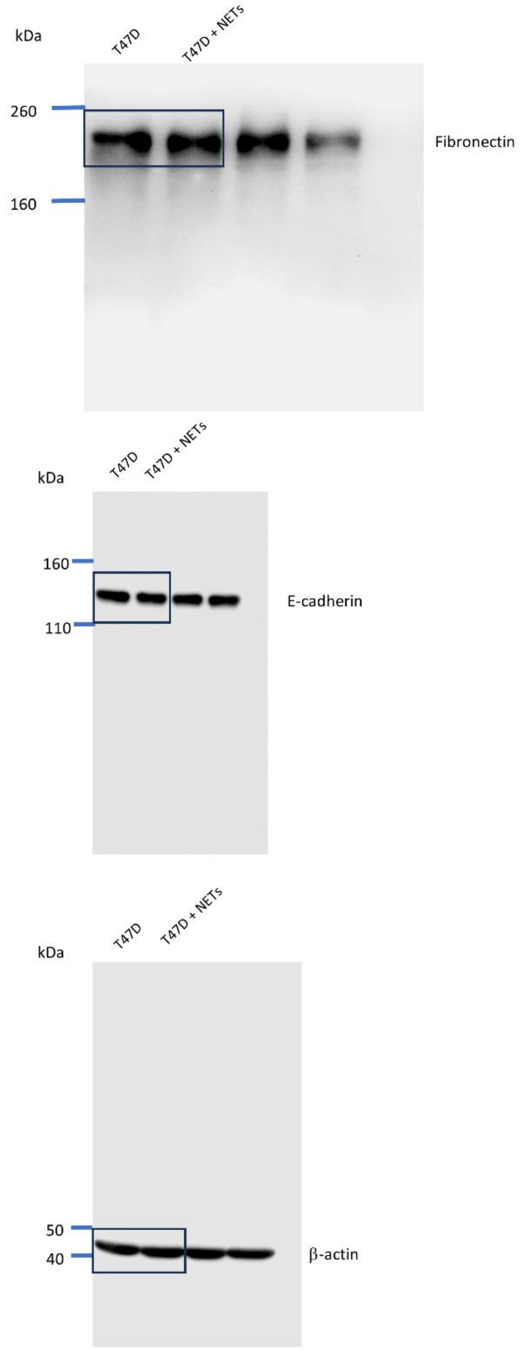

Supplementary Figure S5. Uncropped blots of Figure 7; Fibronectin and E-cadherin staining for T-47D cells
